# Physical Properties of Electropolished CoCrMo Alloy Coated with Biodegradable Polymeric Coatings Releasing Heparin after Prolonged Exposure to Artificial Urine

**DOI:** 10.3390/ma14102551

**Published:** 2021-05-14

**Authors:** Wojciech Kajzer, Janusz Szewczenko, Anita Kajzer, Marcin Basiaga, Joanna Jaworska, Katarzyna Jelonek, Katarzyna Nowińska, Marcin Kaczmarek, Ada Orłowska

**Affiliations:** 1 Department of Biomaterials and Medical Devices Engineering, Faculty of Biomedical Engineering, Silesian University of Technology, 41-800 Zabrze, Poland; janusz.szewczenko@polsl.pl (J.S.); anita.kajzer@polsl.pl (A.K.); marcin.basiaga@polsl.pl (M.B.); marcin.kaczmarek@polsl.pl (M.K.); ada.orlowska@polsl.pl (A.O.); 2 Centre of Polymer and Carbon Materials, Polish Academy of Sciences, 41-819 Zabrze, Poland; jjaworska@cmpw-pan.edu.pl (J.J.); kjelonek@cmpw-pan.edu.pl (K.J.); 3 Department of Applied Geology, Faculty of Mining, Safety Engineering and Industrial Automation, Silesian University of Technology, 44-100 Gliwice, Poland; katarzyna.nowinska@polsl.pl

**Keywords:** metallic biomaterials, CoCrMo alloy, biodegradable polymer coatings, corrosion resistance, artificial urine

## Abstract

In this study, we aimed to determine the effect of long-term exposure to artificial urine on the physical properties of CoCrMo alloy with biodegradable heparin-releasing polymeric coatings. Variants of polymer coatings of poly(L,L-lactide-ɛ-caprolactone) (P(L,L-L/CL)) and poly(D,L-lactide-ɛ-caprolactone) (P(D,L-L/CL)) constituting the base for heparin-releasing (HEP) polyvinyl alcohol (PVA) coatings were analyzed. The coatings were applied by the dip-coating method. Heparin was used to counteract the incrustation process in the artificial urine. The study included tests of wettability, resistance to pitting and crevice corrosion, determination of the mass density of metal ions penetrating into the artificial urine, and the kinetics of heparin release. In addition, microscopic observations of surface roughness and adhesion to the metal substrate were performed. Electrolytically polished CoCrMo samples (as a reference level) and samples with polymer coatings were used for the tests. The tests were conducted on samples in the initial state and after 30, 60, and 90 days of exposure to artificial urine. The analysis of the test results shows that the polymer coatings contribute by improving the resistance of the metal substrate to pitting and crevice corrosion in the initial state and reducing (as compared with the metal substrate) the mass density of metal ion release into the artificial urine. Moreover, the PVA + HEP coating, regardless of the base polymer coatings used, contributes to a reduction in the incrustation process in the first 30 days of exposure to the artificial urine.

## 1. Introduction

A blockage of urinary outflow (obstructive uropathy) can disturb kidney function, as well as lead to urine retention and so-called hydronephrosis. The most common cause of obstructive uropathy is blockage of the ureter, as it is the narrowest part of the urinary excretion system. A blockage can be caused by a urinary stone, ureter cancer, bladder cancer closing the ureteral orifice, a blood clot, as well as tumors in other organs that, when growing, compress the ureter from the outside and close the urethral lumen (colorectal, uterine, or ovarian cancers). Hydronephrosis can cause renal colic, i.e., severe flank pain with nausea and vomiting. Even worse, untreated obstructive uropathy leads to irreversible kidney failure. To prevent this, congestive urine should be drained from the kidney. One method is to set up a patient with a ureteral catheter (or stent) that connects the renal pelvis to the bladder [1,2].

Every year, around 1.5 million stents are implanted worldwide. According to the National Health Fund statistical data from 2012 to 2018, a steady increase in the number of patients receiving urinary tract implants has been observed in Poland (i.e., from 2365 cases of JJ stent implantations, in 2012, to 8332 cases in 2018) [3]. Unfortunately, an increase in the number of stents implanted in the urinary system is accompanied by an increase in the number of complications caused by the procedure as well as the presence of the implant in the body, making it a serious clinical problem. In about 80% of cases, the implant contributes to the occurrence of renal colic, negatively affecting the quality of life, and most often requiring replacement or sometimes surgical intervention [4]. Replacement of the stent with a new one is necessary in the case of persistent pain in the lumbar region, urinary tract infection (bacterial biofilm forms on the stents), vesicoureteral reflux, irritation of bladder tissues (which causes overactive bladder syndrome), or recurring blockage of urine flow due to surface incrustation of the stent [5,6,7].

For years, scientists in the field of biomedical engineering, microbiology, and clinicians have been looking for a way to reduce the side effects of using stents. Research has focused on identifying new materials for the production of stents, including the use of locally releasing active substances to reduce side effects [8]. The purpose of this study is to obtain an implant with a surface material that can limit the formation of bacterial biofilm and, consequently, the incrustation process [9]. 

Materials used to treat the upper urinary tract are divided into polymers and metals. Initially, silicones were used to produce polymer stents due to their low susceptibility to incrustation in non-infected urine. However, incrustation in infected urine was much faster [8,9]. An additional problem was the large coefficient of friction which caused difficulties with its application. Currently, the polyurethanes that are used have better mechanical properties enabling the production of implants characterized by a larger ratio of an implant’s lumen surface to total cross-section. In addition, they are characterized by variable rigidity in the range of room and human body temperatures [4]. Higher stiffness makes it easier to insert an implant into the ureter, at human body temperature, the stiffness is reduced and the implant more easily adapts to the shape of the ureter, reducing undesirable symptoms in the patient. To improve the functional properties of polymer stents, attempts have been made to functionalize their surfaces with active substances [10,11,12,13,14]. 

Alternatively, the materials used for the production of urological implants are metal biomaterials. Their advantage is radial strength and good radiological visibility, while their disadvantage is low deformability, which limits the implant’s adaptation to the anatomy of the ureter [11]. The most commonly used materials are nitinol and cobalt-nickel-chromium-molybdenum alloy.

Clinical studies have shown that metal implants made with metal biomaterials (Co and NiTi alloys [12,13,14]) are less susceptible to biofilm formation, thus, limiting the incrustation process. The average service life of metal stents is three times longer than that of polymer stents. The total costs associated with treatment of an upper urinary tract using metal stents are two times lower as compared with polymer stents. Despite the higher cost of the metal stent itself, its longer residence time that limits the number of procedures performed and therefore patient hospitalization time ultimately leads to a reduction in the total cost of treatment [15,16,17].

Cobalt-based alloys are a group of metal biomaterials used to produce various types of implants, including those used in minimally invasive stenting for systems such as blood and urinary [18,19]. These alloys belong to the group of non-toxic biomaterials [20]. They are characterized by greater biocompatibility than stainless steel resulting from greater corrosion resistance in tissues and body fluid environments. They are also distinguished by greater repassivation abilities in physiological solutions [21]. Their chemical composition and manufacturing conditions both determine their mechanical properties and corrosion resistance [22].

Among the group of cobalt alloys, casting and wrought alloys are distinguished [18,23,24]. The homogeneous and fine-grained structure of wrought alloys as compared with cast alloys results in better resistance to pitting and crevice corrosion [21], and therefore minimally invasive surgeries mostly use wrought alloys.

In the case of mechanically polished cobalt alloys, a passive layer about 2.5 nm thick does not protect the alloy against the aggressive action of chlorine ions contained in body fluids [25]. To improve the corrosion resistance of cobalt alloys subjected to both mechanical and electrolytic polishing, a passivation treatment is used. Chemical passivation is usually performed using nitric acid; however, studies have also been conducted that used sodium salt solutions [26]. Other methods of passivating Co alloys are electrochemical methods [25,27]. Passive layers significantly improve the corrosion resistance of cobalt alloys.

In addition to using oxide layers, there is an alternative method for introducing active substances that could potentially influence the reduction of biofilm formation. It is based on biodegradable polymers, i.e., aliphatic polyesters, which can serve as a matrix for different drugs (so-called biodegradable drug delivery systems). As hydrolytically unstable macromolecular compounds, aliphatic polyesters start to degrade after being placed in a water environment, simultaneously releasing the incorporated drug. The polymers swell and gradually release the active substances by diffusion, as well as due to degradation of the polymeric chain (scissions of the labile bonds, e.g., ester bonds). In fact, biodegradable polymers have become highly important in the field of biomaterials [28,29,30,31]. 

A very popular method for obtaining polymer coatings is dip coating, in which it is possible to obtain a polymeric layer with different thicknesses. Recently, ultrasonic spraying is a method that has been used, which involves the formation of fine droplets of a coating solution and their deposition on a substrate [32,33,34]. 

The process of spontaneous deposition of extracellular matrix proteins and bacteria occurs on the surface of biomaterial in contact with tissues and urine. The composition and amount of material deposited on the surface depend on the biological reactivity of the biomaterial which depends on the following: the chemical composition of the surface, its morphology, wettability (surface energy), and electrical properties. Embedded proteins affect the processes of cell and bacterial adhesion, cell biological activity, and the activation of inflammatory reactions. Knowledge of the reaction mechanisms occurring on the biomaterial surface in contact with tissues enables targeted modification of the biomaterial surface layer aimed at limiting or eliminating the biofilm formation process, which is the initial stage in the development of the incrustation process [35,36,37,38,39,40].

Therefore, in this study, we investigated the usefulness of surface modification of the CoCrMo alloy, intended for urological implants, by applying biodegradable polymer coatings that release active substances to reduce incrustation. The research included preparation of the metal substrate and application of different types of biodegradable polymer coatings to which an outer coating containing the active ingredient (heparin) was applied. For the tests on samples prepared in this way, the impacts of 30-, 60-, and 90-day exposures to artificial urine on the physical properties of the modified metal biomaterial and the degradation process of the applied coatings were assessed.

The positive results of the long-term exposure of coatings to artificial urine may be the basis for the development of stents with a longer service life without the need to replace them.

## 2. Materials and Methods

### 2.1. Surface Preparation

The CoCrMo alloy, which fulfills the requirements of ISO 5832-12, was used for the tests. The tests were conducted on samples in the form of rods with a diameter of 6 mm and a length of 50 mm, a diameter of 6 mm and a length of 100 mm, and discs with a diameter of 25 mm and a thickness of 3 mm. In the first stage, the metal substrate was prepared for testing; the surface of the alloy was ground with 500 and 1000 grit sandpaper to remove the hardened layer of material remaining from the preparation of the sample form. Next, the samples were electrolytically polished to obtain a surface roughness below Ra < 0.16 µm and to make the surface glossy. For the electropolishing process, a commercial CHEMIA-ELEKTROMETR Elektrol I solution was used, which included ethylene glycol, sulfuric acid, and hydrochloric acid. Polishing was carried out at a temperature of 35 °C and a current intensity of 2 A for 7 min. Polymer coatings were applied to the prepared surfaces. Two types of base coatings were used: poly(L,L-lactide-ɛ-caprolactone) (P(L,L-L/CL)) and poly(D,L-lactide-ɛ-caprolactone) (P(D,L-L/CL)). Copolymers, i.e., poly(L,L-lactide-ɛ-caprolactone) and poly(D,L-lactide-ɛ-caprolactone) were obtained by ring-opening polymerization (ROP) of L,L-lactide or D,L-lactide with ɛ-caprolactone, in the presence of zirconium (IV) acetylacetonate as an initiator. From the synthesized polymers, solutions with a percentage concentration of Cp = 1% were prepared using dichloromethane CH_2_Cl_2_ as a solvent. Due to the poor solubility of heparin in organic solvents and the consequent inability to obtain a homogeneous solution of heparin in P(L,L-L/CL) and P(D,L-L/CL), an additional outer layer of heparin-containing polyvinyl alcohol (PVA) was produced. The outer layer was comprised of a 5% aqueous PVA solution. The heparin content in the PVA coating was 20% (*w*/*w*). The base and heparin-releasing coatings were applied using the dip method using a dip coater (PTL-OV6P, MTI Corporation, Richmond, VA, USA). One immersion cycle lasting 30 s was used. After 5 days of drying under vacuum conditions, samples coated with P(D,L-L/CL) or P(L,L-L/CL) were immersed, using the same apparatus, in a PVA + HEP solution. The immersion time was equal to 60 s, for one cycle. In this way, biodegradable coatings on Co-alloy were obtained, consisting of P(D,L-L/CL) + PVA + HEP and P(L,L-L/CL) + PVA + HEP polymer compositions.

The obtained samples were divided into a reference group (CoCrMo alloy substrate and samples with coatings) and groups subjected to 30, 60, and 90 days of exposure to artificial urine. The long-term studies were aimed at checking the suitability of using the proposed coatings as an alternative to the present standard, which is a polymer stent replaced every 30 days. During the exposure, a stand was used to simulate the daily urine flow (0.750 dm^3^) through one ureter. The stand was placed in a Qcell500/50 laboratory incubator by POL-LAB (Pol-Lab Sp. Z. o.o., Wilkowice, Poland), ensuring a constant temperature of 37 °C. Artificial urine with a chemical composition compliant with ASTM 1828—93 [41] (pH = 5.3–6.3) was used for the tests: NaCl, 6.17 g/dm^3^; NaH_2_PO_4_, 4.590 g/dm^3^; Na_3_C_6_H_5_O_7_, 0.944 g/dm^3^; MgSO_4_, 0.463 g/dm^3^; Na_2_SO_4_, 2.408 g/dm^3^; KCl, 4.750 g/dm^3^; CaCl_2_, 0.638 g/dm^3^; and Na_2_C_2_O_4_, 0.043 g/dm^3^. All ingredients were dissolved in demineralized water.

### 2.2. Microscopic Observations

Observations of the samples’ surfaces were performed using a Zeiss SteREO Discovery.V8 stereoscopic microscope with an AxioVision camera (Zeiss, Oberkochen, Germany), and a scanning electron microscope (SEM) (Quanta 250 FEG, Thermo Fisher Scientific, Hillsboro, OR, USA). The samples were observed at the initial state and after exposure to artificial urine.

### 2.3. Surface Roughness

The surface roughness was measured on the elemental section lc = 0.8 mm, in accordance with the recommendations of PN-EN ISO 1302:2004 [42] using a SURTRONIK S128 contact profilometer by Taylor Hobson (Taylor Hobson Ltd., Leicester, UK). For each test sample, 6 measurements were made, and the arithmetic mean of the Ra profiles was determined. The tests were conducted on samples in their initial state.

### 2.4. Wettability Tests

The wettability test was performed using a SURFTENS UNIVERSAL goniometer by OEG (OEG, Frankfurt, Germany) equipped with a digital camera and a computer with Surftens 4.5 Software for the analysis of the recorded image. The contact angle measurements were made using demineralized water on discs with a diameter of 25 mm. The samples were tested in the initial state and after 30, 60, and 90 days of exposition to the artificial urine. Five drops of a volume of 1 mm^3^ each were placed successively on the surface of each sample and the measurement was started after 15 s. The duration of a single measurement was 60 s with a sampling frequency of 1 Hz. The test was conducted at room temperature T = 23 ± 1 °C. The mean contact angle, θ_av_, for water was determined. The analysis was performed on the samples at their initial state and after exposure to artificial urine.

### 2.5. Adhesion Test

In order to evaluate the influence of artificial urine on the adhesion of the produced polymer coatings, an adhesion test was performed. The tests on the adhesion of the P(L, L-L/CL) and P (D,L-L/CL) coatings to the substrate in the initial state and after 30, 60, and 90 days of exposure to the artificial urine were conducted on disc-shaped samples with a diameter of 25 mm utilizing the scratch test method, using an open platform equipped with a MicroCombi Tester by CSM (Anton Paar GmbH, Graz, Austria). A diamond cone (Rockwell, Anton Paar GmbH, Graz, Austria) indenter was used for the test. A gradually increasing loading force was applied in the range of 0.03 ± 30 N. The table travel speed was 1.0 mm/min, the table load rate was 10 N/min, and the scratch length was 3 mm. The critical force, F_n_, was assessed based on the registered changes in the friction force as a function of the scratch length of the analyzed polymer coatings. The reference sample was a sample without a polymer coating and not exposed to the artificial urine. The tests were conducted on the samples in their initial state and after 30 days of exposure to the artificial urine. Three scratches were made on each of the tested samples.

### 2.6. Potentiodynamic Corrosion Test

Pitting corrosion resistance was tested using the potentiodynamic method, based on the recording of polarization curves. A VoltaLab PGP 201 potentiostat with VolaMaster 4 software (Radiometer, Villeurbanne Cedex, France) was used for the study. The research was conducted with artificial urine. A silver chloride electrode (Ag, AgCl/3M KCl) was used as the reference electrode, and a platinum (Pt) electrode was used as the auxiliary electrode. From each variant, 3 rod-shaped samples with a length of 50 mm were tested. The measurements were started by determining the value of the open circuit potential, E_ocp_, and then the polarization curves were recorded. The recording of polarization curves was started from the E_init_ potential = E_ocp_ – 100 mV. The potential change took place in the anode direction at a rate of 3 mV/s. After reaching the limiting current density of 1 mA/cm^2^, the direction of polarization was changed, and the return curve was recorded. On the basis of the obtained diagrams, the values of parameters characterizing the resistance to pitting corrosion were determined, i.e., the corrosion potential, E_corr_, the transpassivation potential, E_tr_, and the polarization resistance, R_p_. The corrosion potential and the polarization resistance were both determined using the Stern method. The tests were conducted in the artificial urine on the samples in the initial state and after 30, 60, and 90 days of exposure to the artificial urine.

### 2.7. Potentiostatic Corrosion Test

The crevice corrosion resistance test was performed using the potentiostatic method, recording the curves of the current density as a function of time. The same measuring stand was used for the test as in the potentiodynamic tests. The measurements were started with the determination of the value of the open circuit potential E_ocp_. Then, a potentiostatic curve was recorded, based on which the surface charge density, Q, was determined and, based on its course, the crevice corrosion resistance was assessed. The change of current density in time t = 15 min was recorded for potential E = 800 mV in accordance with the recommendations of ASTM F745-04:2009 [43]. The crevice corrosion resistance tests were conducted on samples in the form of a 50 mm long bar in the initial state and with coatings of biodegradable polymers. The tests were performed for 3 samples from each variant. The tests were conducted in the artificial urine for the initial state samples and after 30, 60, and 90 days of exposure to the artificial urine.

### 2.8. Investigation of the Mass Density of Ions Released into the Solution

The JY 2000 spectrometer by Jobin-Yvon (Jobin-Yvon Horiba, Kyoto, Japan) was used for the analysis of the mass density of ion release into the solution. The ICP-AES plasma atomic emission spectrometry method was used. The tests were conducted on samples without coatings and on samples with coatings at 0, 30, 60, and 90 days of exposure. The obtained results were converted into the surface mass density of metal ions (Co, Cr, Mo) released into the artificial urine.

### 2.9. Examination of Surface Degradation and Incrustation

The degradation and incrustation processes were assessed based on macroscopic observations and the mass balance of samples before and after exposure to artificial urine. The tests were conducted on samples in the form of 100 mm long rods without coating and with coatings after 30, 60, and 90 days of dynamic exposure. The mass measurement was performed on a laboratory balance AKA320 by AXIS (AXIS Sp.z o.o., Gdańsk, Poland) using 3 samples for each analyzed variant of surface modification.

### 2.10. Investigation of the Kinetics of the Heparin Release Process

Samples of CoCrMo alloy in the form of 50 mm long bars coated with the biodegradable polymers were immersed for 21 days in deionized water to study the heparin release process. The samples were kept at 37 °C under agitation (120 rpm). After sampling at the predetermined intervals, the water was replaced to maintain the sink condition. The medium was collected at designated times and the heparin concentration was analyzed using the spectrophotometric method [44,45] based on the interaction of Azur A dye with heparin. As a result, the solution with an initially intense blue color changes its color and the intensity of the color is proportional to the number of heparin molecules that react with the dye. For the spectrophotometric measurements, an Azur A solution with a concentration of 8 µg/mm^3^ was prepared and used for the determination of heparin: 100 mm^3^ of Azur A solution was added to 100 mm^3^ of the medium taken and mixed by vortexing for 15 min. Measurement was performed at 535 nm using a Tecan Spark multi-sensing plate reader (Tecan Group Ltd. Seestrasse (Männedorf), Switzerland). The intense blue color of Azur changed to purple or magenta. In order to determine the amount of released heparin, in the first step, a curve of heparin concentration versus absorbance value was developed. For this purpose, several heparin concentrations in water were made. For a series of solutions with known heparin concentration, the absorbance value was measured at a wavelength of 535 nm. 

### 2.11. Statistical Analysis

The physical and electrochemical test results were presented as means with standard deviation. In order to determine the significance of differences for *p* < 0.05, the obtained results used a one-way analysis of variance (ANOVA).

## 3. Results and Discussion

### 3.1. Results of Microscopic Observations

**Figure 1:**
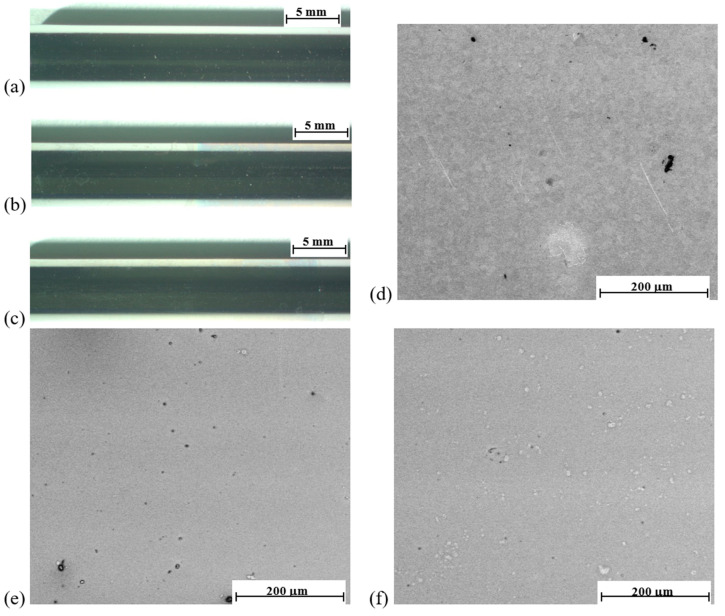
Observations of the samples’ surfaces in the initial state. Stereoscopic microscope of cylinder-shaped samples. (**a**) Electropolished surface; (**b**) electropolished with P(L,L-L/CL) + PVA + HEP coatings; (**c**) electropolished with P(D,L-L/CL) + PVA + HEP coatings. Scanning microscope of samples in the shape of discs. (**d**) Electrolytic polished surface; (**e**) electropolished with P(L,L-L/CL) + PVA + HEP coatings; (**f**) electropolished with P(D,L-L/CL) + PVA + HEP coatings.

The microscopic observations of the samples’ surfaces showed that both types of polymer coatings were characterized by transparency, continuity, and homogeneity (Figure 1). Similar features of polymer coatings, but with different chemical compositions, applied with the dip-coating method on a substrate made with metal biomaterials have been shown by authors in their earlier works [46,47,48,49,50,51,52,53].

### 3.2. Surface Roughness Test Results

The mean values of the Ra parameter together with the standard deviation for the samples without and with polymer coatings are summarized in Table 1. The analysis of the obtained data showed that the roughness of electrolytically polished samples was similar to the roughness of the produced coatings. This proves that the topography of the metal substrate is inherited by the coatings.

### 3.3. The Results of the Surface Wettability Tests

**Figure 2:**
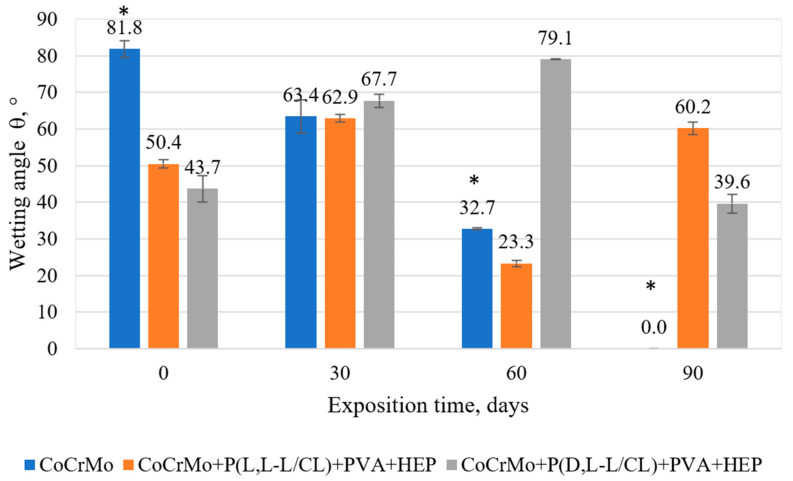
Values of the contact angle for the tested variants of surface modification (* *p* < 0.05 versus the control group).

**Figure 3:**
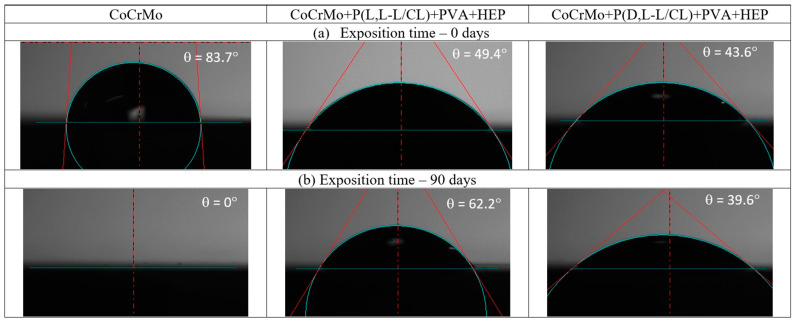
Exemplary figure of the drops during the wettability test. (**a**) Initial state (0 days of exposition); (**b**) after 90 days of exposition in artificial urine.

According to the obtained results (Figure 2 and Figure 3), different degrees of surface wettability were found depending on the applied coating and the time of exposure to the artificial urine. The lowest wettability was obtained for the electrolytically polished CoCrMo alloy without applied coatings. In turn, the compositions of biodegradable polymer coatings applied to the metal substrate significantly increased the wettability of the surface (*p* < 0.05) as compared with the uncoated samples. The wettability of the surface has a significant influence on the adhesion, proliferation, and growth of cells on the surface of the tested biomaterials [54].

Exposure of the samples to the flow of artificial urine caused various changes in the wettability of the tested samples. This was due to two processes that opposed the surface wettability. The increase in wettability could be due to the hygroscopic properties of the sediment observed on the surface of the test samples after exposure to artificial urine. On the one hand, for the uncoated samples, complete wettability was observed after 90 days of exposure. On the other hand, for samples with polymer coatings, the increase in hydrophobic properties after 30-day exposure may be related to the presence of caprolactone segments showing hydrophobicity [55] from the base coatings exposed after partial degradation of the outer PVA + HEP coating. The authors found a similar ambiguity in the changes in the value of the contact angle for biodegradable polymer coatings during exposure to Ringer’s solution [50].

### 3.4. Results of Tests of Coating Adhesion to the Substrate

**Figure 4:**
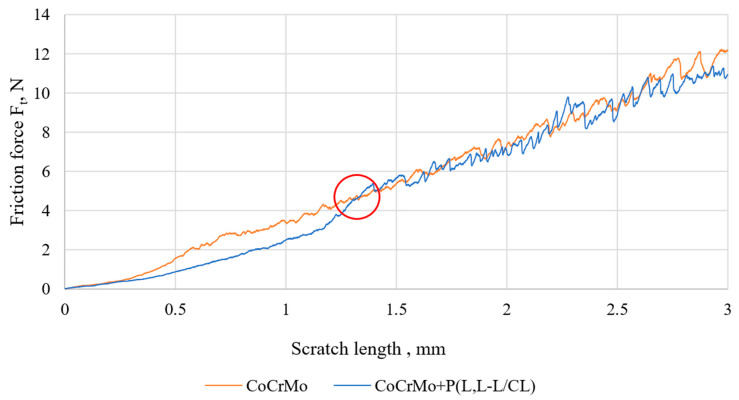
An exemplary graph of the friction force as a function of the scratch length of uncoated and P(L,L-L/CL) coated samples.

The critical force illustrating the adhesion of the polymer coating to the metal substrate was determined by comparing the friction force courses as a function of the displacement of the metal substrate sample and the samples with the applied polymer coatings. The value of the force at the intersection of the diagrams (Figure 4) was taken as the adhesion force of the polymer coating to the metal substrate. After reaching this value, a similar course of the graphs is observed, which is the result of recording the friction force coming from the metal substrate. A similar methodology for determining the value of the critical force of the tested polymer coatings on a metal substrate was determined by the authors [49,51].

The P(D,L-L/CL) coating (F_n_ = 5.92 N, Table 2) was characterized by the highest critical force among the base coatings. The analysis of the obtained results shows that regardless of the type of biodegradable base coating, the values of the critical force are similar, and they increase slightly after 30 days of exposure to artificial urine. Similar values of the critical force, both in the initial state and after 30 days of exposure in Ringer’s solution, were found for coatings with poly(glycolide-ɛ-caprolactone) and poly(glycolide-ɛ-caprolactone-L,L-lactide) applied to the anodically oxidized substrate with Ti6Al7Nb alloy [51]. In addition, irrespective of the type of polymer coating, further (60 and 90 days) exposure to the artificial urine solution decreased the value of the critical force Fn.

### 3.5. Results of Potentiodynamic Tests of Resistance to Pitting Corrosion

**Figure 5:**
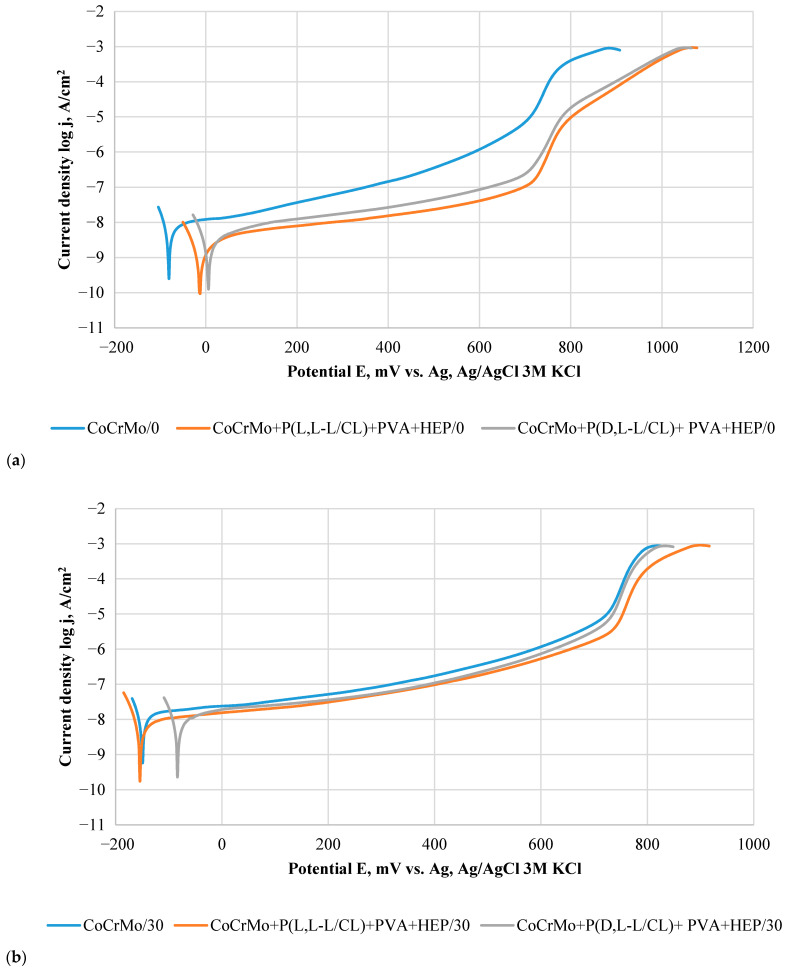

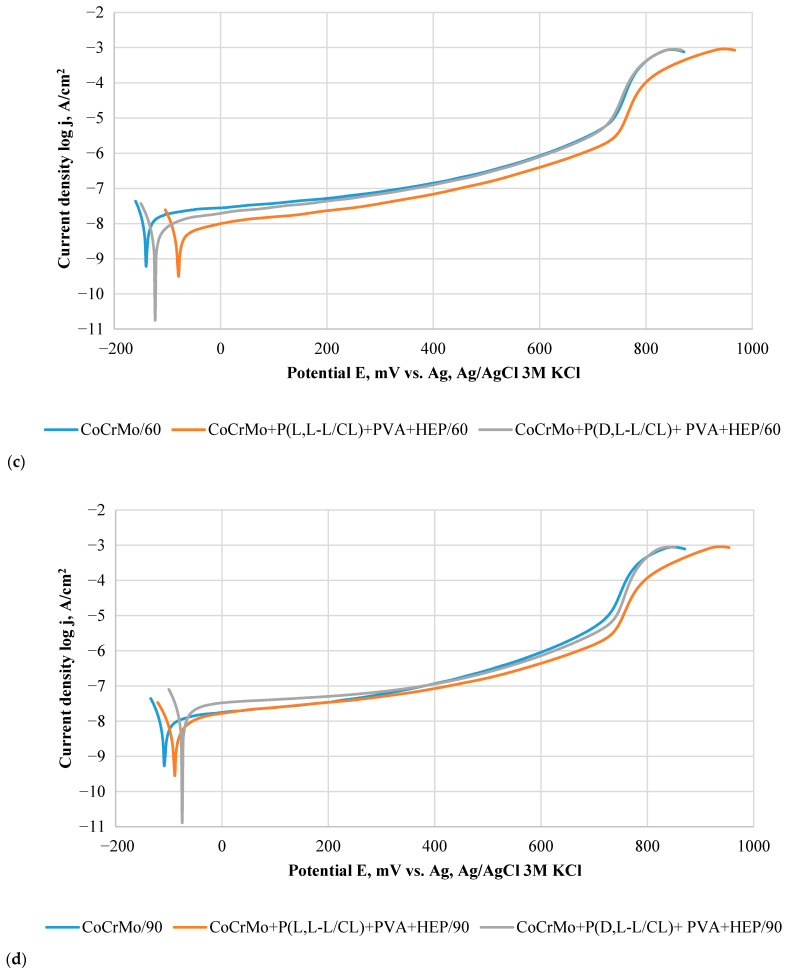
Exemplary polarization curves for the tested variants of CoCrMo alloy surface modification. (**a**) Initial state; (**b**) after 30 days; (**c**) after 60 days; (**d**) after 90 days, of exposure to artificial urine.

In the course of the potentiodynamic curves (Figure 5), regardless of the type of samples (initial state, and covered with biodegradable coatings), there was a potential for transpassivation, which proves the resistance of the tested materials to pitting corrosion. The values of corrosion potential and transpassivation, as well as polarization resistance determined on the basis of the curves, are summarized in Table 3.

The application of polymer coatings resulted in a slight increase in the corrosion potential and a significant (*p* < 0.05) increase in the value of the E_tr_ transpassivation potential, which for the metal substrate was 790 mV, while for the samples with polymer coatings it was about 960 mV. The application of polymer coatings also caused a significant (*p* < 0.05) three-fold increase in the polarization resistance and a decrease in the current density in the passive range (Figure 5a). A similar positive effect of biodegradable polymer coatings on pitting corrosion resistance of a metal substrate has been confirmed by the authors’ of [46,47,48,50]. Exposure to artificial urine decreased the value of the corrosion potential for all types of samples. A similar direction of changes was observed for the value of the transpassivation potential in the case of samples with polymer coatings. For samples without polymer coatings, no significant (*p* > 0.05) effect of long-term exposure to artificial urine on the value of the transpassivation potential was observed. Similar results were obtained by the authors of [56,57] for tests on the corrosion resistance of Co alloys conducted in Ringer’s solution, PBS, and NaCl solution. Regardless of the duration of exposure to artificial urine and the type of tested samples, similar current density values were found in the passive range (Figure 5b–d). The value of the polarization resistance of the samples, regardless of the type of surface, as a result of exposure to artificial urine, decreased as compared with the initial state (Table 3). In the case of P(L,L-L/CL) + PVA + HEP, the polarization resistance value was constant over time and amounted to about 2 MΩ⋅cm^2^, while for P(D,L-L/CL) + PVA + HEP, a decrease in the value of polarization resistance was observed.

### 3.6. Results of Potentiostatic Tests of Resistance to Crevice Corrosion

**Figure 6:**
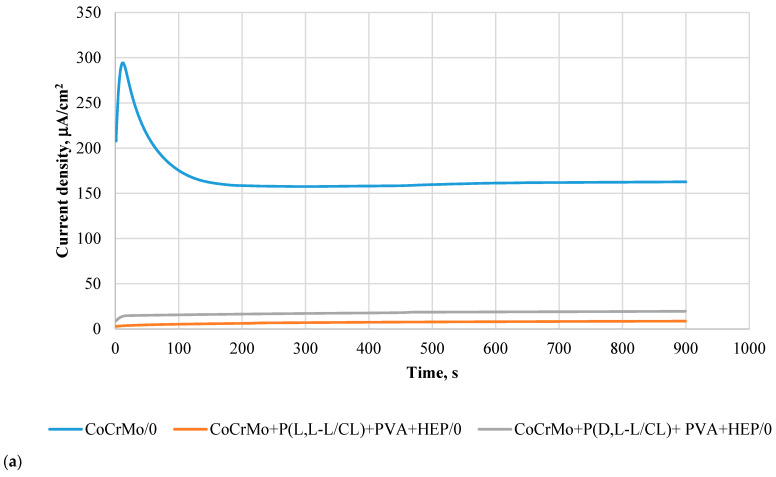

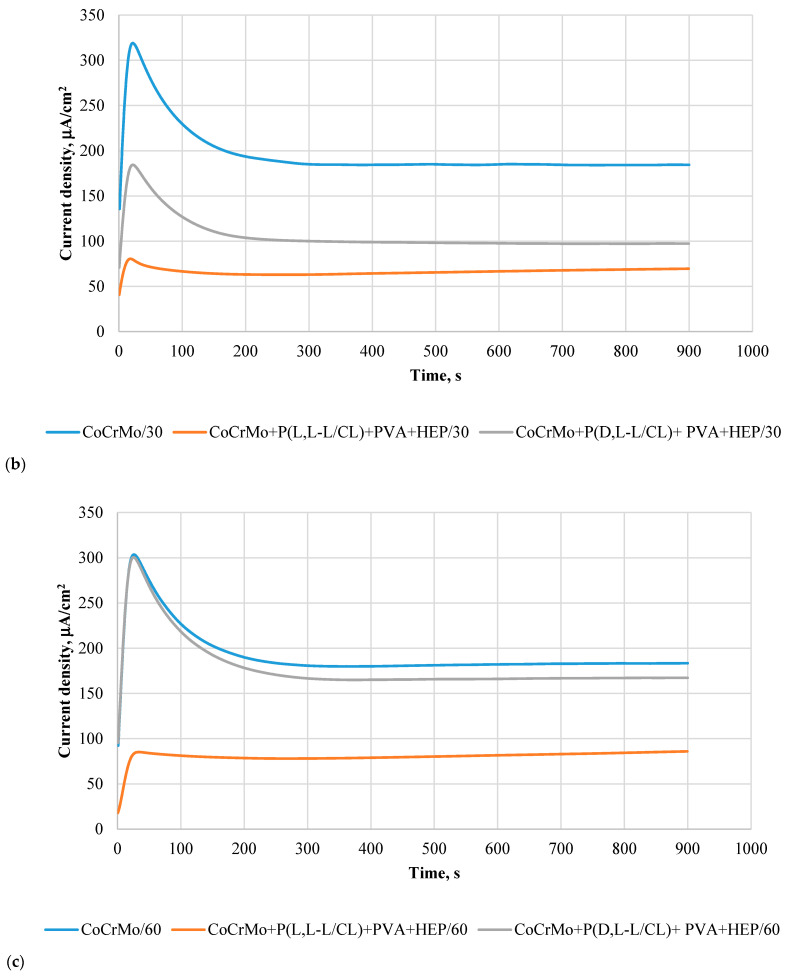

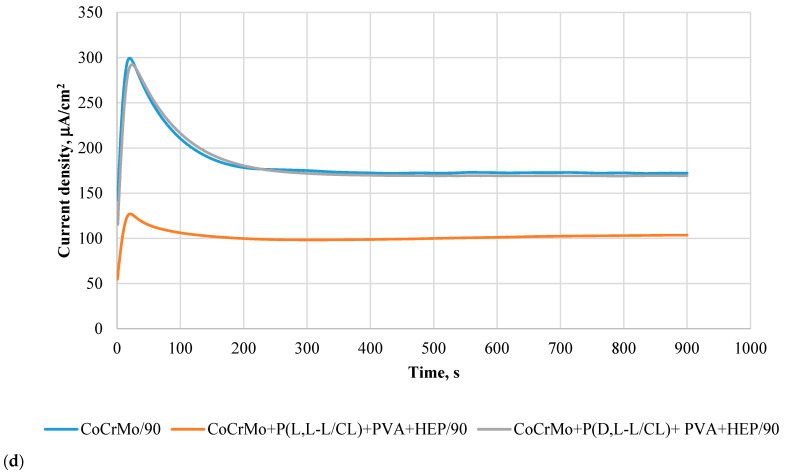
Comparison of exemplary potentiostatic curves for E = 800 mV for the tested variants. (**a**) In the initial state, (**b**) after 30 days; (**c**) after 60 days; (**d**) after 90 days, of exposure to artificial urine.

**Figure 7:**
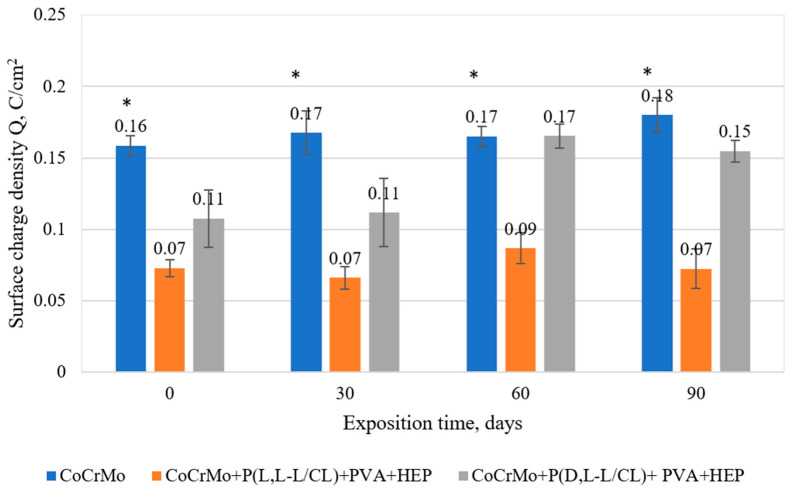
Comparison of mean values of electric flux density determined in potentiostatic studies for the tested variants of CoCrMo alloy surface modification, with exposure time to artificial urine up to 90 days (* *p* < 0.05 versus the control group).

The course of the potentiostatic curves, regardless of the tested sample surface variants, was characterized in the initial period (15 s) by an increase in the current density, and then its decrease (Figure 5a–d). The recorded curves prove the resistance of the tested samples to crevice corrosion, at the same time indicating the beneficial effect of the applied polymer coatings. Detailed analysis of the current density shows that its value depends on the type of coating and the period of exposure to the artificial urine. In the initial state, the lowest current density values were observed for the samples covered with the P(L,L-L/CL) + PVA + HEP coatings. For all types of surfaces, an increase in the current density was observed over the exposure time, however, the lowest values still characterized the P(L,L-L/CL) + PVA + HEP coatings (Figure 6a–d). In the case of the P(D,L-L/CL) + PVA + HEP coatings, a progressive increase in the current density was observed, after 90-day exposure, the recorded current density was similar to that characterizing electrolytically polished Co alloy substrate. These observations are confirmed by the determined values of the surface charge density, Q, calculated for the determined curves (Figure 7).

### 3.7. The Results of the Analysis on the Mass Density of Ions Released into the Solution

**Figure 8:**
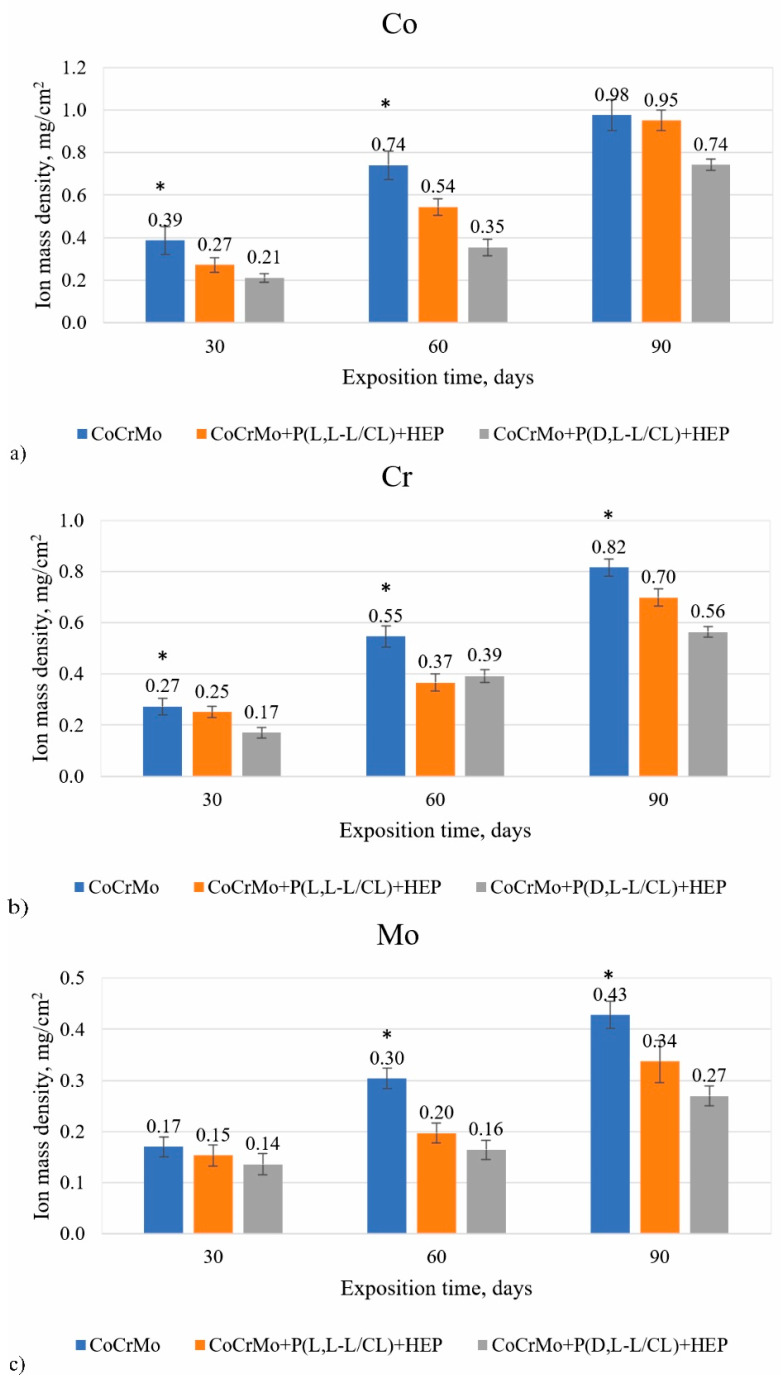
Comparison of the mass density of ions released into the artificial urine for (**a**) cobalt; (**b**) chromium; (**c**) molybdenum (* *p* < 0.05 versus the control group).

The analysis of the chemical composition of artificial urine after 30-, 60-, and 90- day exposure of electrolytically polished CoCrMo alloy with polymer coatings showed that the coatings constituted a barrier limiting the penetration of metal ions from the substrate (Co, Cr, and Mo, Figure 8a–c). Studies have found a positive effect of the application of biodegradable polymer coatings on a metal substrate, that limited or even blocked the penetration of metal ions into the corrosive solution [46,47,48,50].

In particular, we found that, regardless of the type of coating, the applied polymer coatings contributed to a significant (*p* < 0.05) decrease in the mass density of Cr and Mo ions released into the artificial urine, in particular after 60 and 90 days of exposure. On the one hand, in the case of Co ions for the P(D,L-L/CL) + PVA + HEP coatings, also up to 90 days of exposure, a limitation of its release into the solution is ensured. On the other hand, for the variant of Co alloy surface modification with the P(L,L-L/CL) + PVA + HEP coatings, the release of Co ions after 90 days of exposure is similar to that recorded for electrolytically polished Co alloy.

### 3.8. The Results of the Surface Degradation and Incrustation Studies

**Figure 9:**
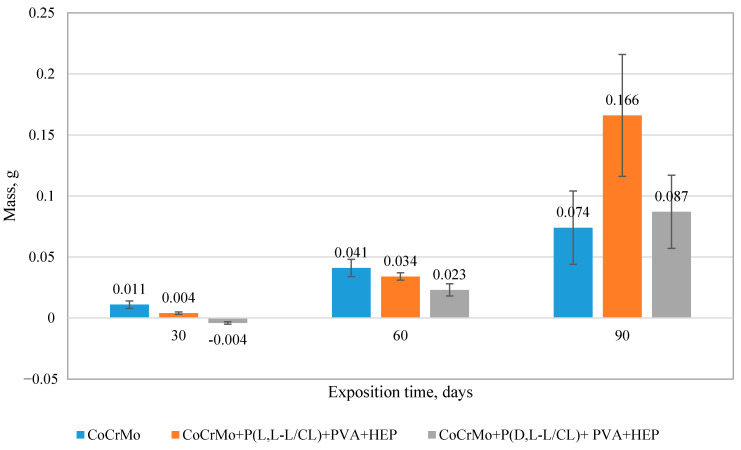
Mass balance of samples (with and without heparin coatings) subjected to 90-day dynamic exposure to artificial urine (*p* < 0.05 versus the control group).

Exposure to the artificial urine of Co-alloy samples with polymer coatings showed that these coatings contributed to a reduction in the incrustation process, as demonstrated by the observations of the samples and the analysis of the results of the sample mass balance tests. The weight gains of the samples caused by the encrustation process with mineral compounds derived from the artificial urine are shown in (Figure 9). The most favorable effect of polymer coatings was observed for exposure up to 60 days. Further exposure (up to 90 days) showed that the incrustation intensified and occurred on all analyzed sample variants. The highest weight gain was found for the samples coated with P(L,L-L/CL) + PVA + HEP.

**Figure 10:**
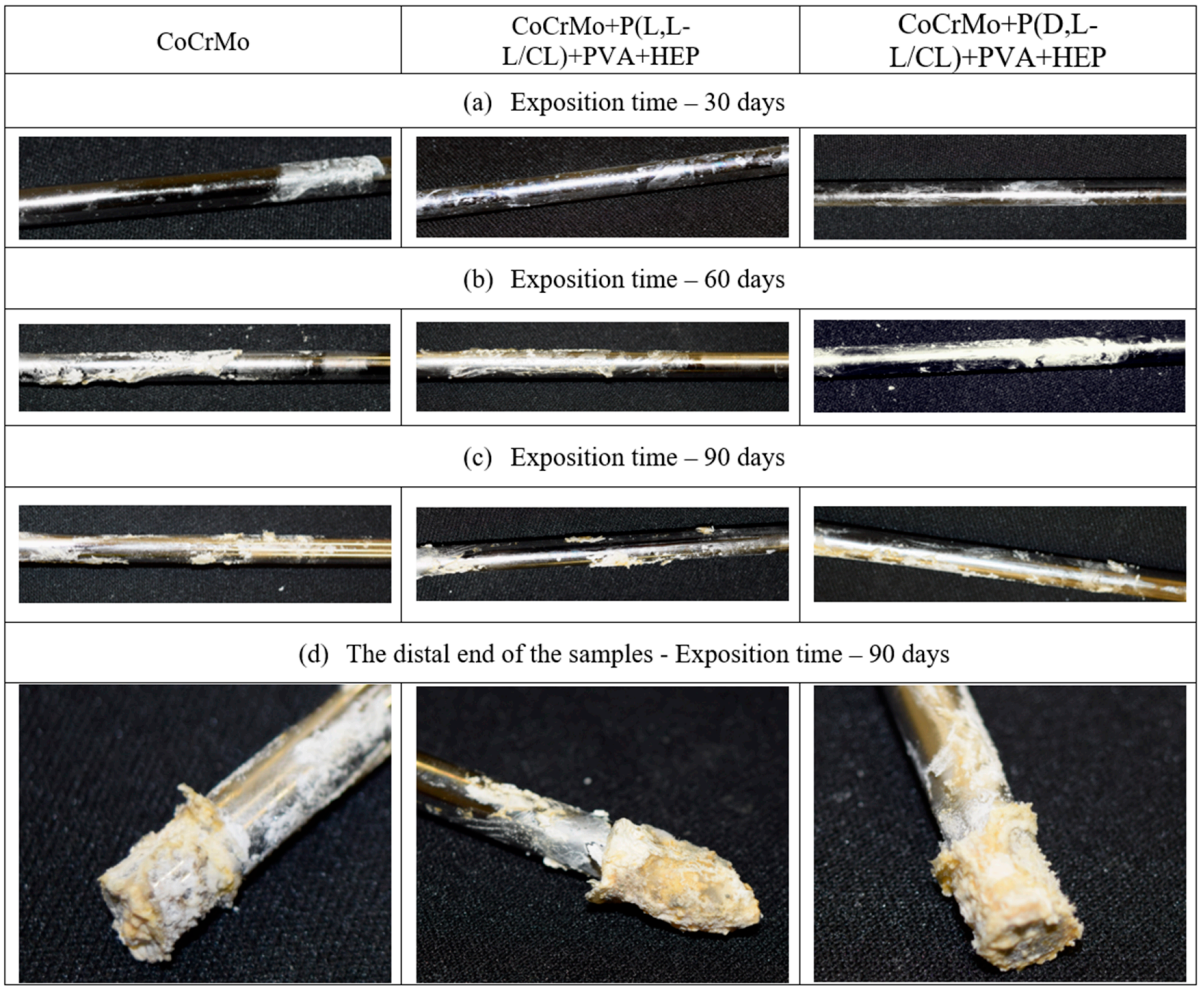
Examples of surfaces of test specimens with visible incrustation after 30, 60, and 90 days of exposure to artificial urine. Samples after exposition: (**a**) 30 days; (**b**) 60 days; (**c**) 90 days; (**d**) 90 days—distal end of the samples.

Macroscopic analysis of the surface of the samples exposed to the artificial urine (Figure 10a–c) showed surface incrustation, the amount of which depended on the exposure time and the type of surface modification applied. In particular, we found that on the samples with biodegradable polymer coatings there was a slight bloom, while, on the uncoated samples, visible clusters of incrustation were clearly visible. Regardless of the surface type, after 90 days of exposure to the artificial urine, a significant increase in the mass of the samples was observed due to the accumulation of sediment at their ends (Figure 10d).

### 3.9. The Results of the Studies on the Kinetics of the Heparin Release Process

**Figure 11:**
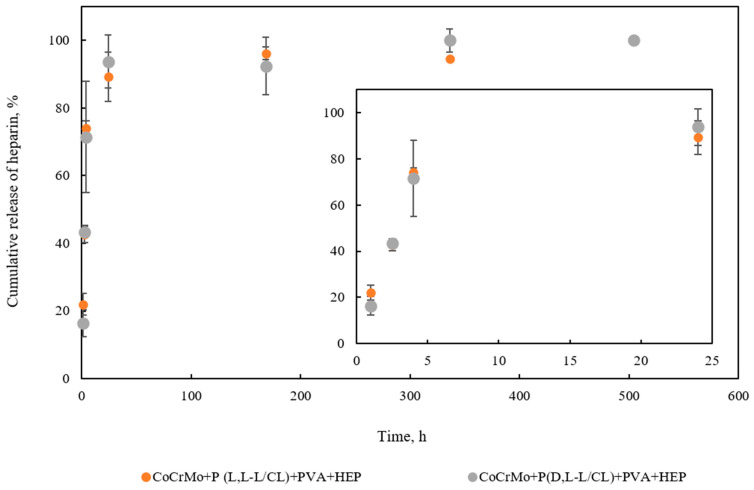
In vitro cumulative heparin release from polymer coatings over 504 h.

The kinetics of heparin release from the PVA + HEP coating applied to the CoCrMo alloy modified with polymer coatings of P(L L-L/CL) and P(D,L-L/CL) are characterized by a different course (Figure 11). A linear relationship of heparin release was observed during the first 24 h. After this period, a significant slowdown in the heparin release process from the coating was observed. The complete release of heparin occurred within 504 h. The profile of heparin release from the biodegradable coatings applied to the CoCrMo is presented in Figure 10. The burst of the drug was detected within 5 h of incubation. During 24 h, nearly 90% of loaded heparin was released. Then, the remaining heparin was released in a slower manner and 100% of heparin was released within 504 h.

## 4. Conclusions

In this study, we aimed to determine the effect of polymer coatings of poly(L,L-lactide-ɛ-caprolactone) (P(L,L-L/CL)) and poly(D,L-lactide-ɛ-caprolactone) (P(D,L-L/CL)), forming a substrate for heparin-releasing polyvinyl alcohol (PVA) (HEP) coating, on the physical properties of an electropolished CoCrMo alloy.

The polymer coatings produced on the Co alloy surface are transparent, continuous, and homogeneous. The surface topography tests showed a very good representation of the metal substrate by the produced coatings. The polymer base coatings showed good adhesion to the substrate. The critical force value F_n_ was about 5 N for the P(L,L-L/CL) coating and about 6 N for the P(D,L-L/CL) coating and did not change significantly after 30 days of artificial urine exposure. The outer PVA + HEP coating, regardless of the type of the base coating, was characterized by greater hydrophilic properties (contact angle θ was about 50°) as compared with electrolytically polished Co alloy (contact angle θ about 80°). Exposure to artificial urine caused ambiguous changes in the wettability of the tested samples. For samples without coatings, after 90 days of exposure to artificial urine, the incrustation sediment formed led to complete wettability of the tested alloy surface. However, in the case of samples with polymer coatings, there were no unequivocal trends in wettability changes. The wettability was affected by the incrustation products and by the degradation of the outer PVA + HEP coating, which resulted in the appearance of caprolactone segments with hydrophobic properties from the base coatings.

The produced polymer coatings, regardless of their type, increase the corrosion resistance of the electrolytically polished Co alloy. The increase in pitting corrosion resistance of samples with polymer coatings is evidenced by the increase in the value of the E_tr_ transpassivation potential and the polarization resistance R_p_ as compared with the electrolytically polished samples. After 90 days of exposure to artificial urine, the E_tr_ and R_p_ parameters, characterizing the coated and electrolytically polished samples, reach similar values. This proves the beneficial effect of polymer coatings that, for up to 90 days, contribute to the improvement of the corrosion resistance of the substrate, while after their degradation, they do not adversely affect the resistance of the substrate. The recorded decrease in the surface charge density, Q, of samples with polymer coatings as compared with the electrolytically polished substrate proves the beneficial effect of the coatings on the resistance to crevice corrosion. Along with the exposure time, the surface charge density for the samples coated with polymers approached the values obtained for the uncoated samples. Overall better pitting and crevice corrosion resistance was observed for the samples with the P(L,L-L/CL) + PVA + HEP coatings.

Polymer coatings limit the process of metal substrate degradation, which resulted in a lower mass density of ions (Co, Cr, Mo) released into the artificial urine as compared with electrolytically polished samples. The composition of polymers of the P(D,L-L/CL) + PVA + HEP coatings was characterized by more favorable barrier properties for the penetration of degradation products of the metal substrate.

In the initial period (up to 30 days), a clearly beneficial effect of polymer coatings on the process of limiting the encrustation of samples exposed to artificial urine was observed. With the lapse of exposure time, microscopic observations and the mass change balance indicate a reduction in this beneficial process. After 90 days of exposure to artificial urine, a similar incrustation was observed on the tested samples with polymer coatings and electrolytically polished substrate.

The analysis of the kinetic results of heparin release from the PVA + HEP coating showed that this process takes place within 24 h at a constant rate. After this time, it slows down considerably to achieve complete release of heparin from the coating after 504 h (about 20 days). The reduction in the incrustation process of the coatings in the first 30 days of exposure to the artificial urine proves the beneficial effect of heparin release from the polymer coating. This beneficial phenomenon may help to reduce the number of complications following implantation of cobalt urological stents.

The conducted tests clearly confirm that the applied coatings constitute a good protective barrier, contributing to the improvement of the corrosion resistance of the metal substrate and the reduction in corrosion products penetrating into the artificial urine. An in-depth analysis of the physical properties of the tested coatings does not allow for an unambiguous statement regarding which coating has better properties.

The analysis of the results clearly indicates the advisability of further work on the modification of metal biomaterials with biodegradable polymer coatings that release active substances to reduce the incrustation process. The positive results of these studies should, in clinical practice, contribute to the extension of the time of using metal implants, which will have a positive effect on the patient’s comfort. In addition, it is important to reduce treatment costs by reducing the number of expensive stent replacement procedures, as well as to reduce the number of systemic drugs consumed by patients.

## Figures and Tables

**Table 1:** Results of roughness measurements.

Sample	Ra, µm
CoCrMo	0.11 ± 0.02
CoCrMo+P(L,L-L/CL) + PVA + HEP	0.12 ± 0.02
CoCrMo+P(D,L-L/CL) + PVA + HEP	0.12 ± 0.02

**Table 2:** Summary of test results for the adhesion of P(L,L-L/CL) and P (D,L-L/CL) coatings to the substrate.

Sample	Critical Force F_n_ (N) after Exposition Time			
	0 Days	30 Days	60 Days	90 Days
CoCrMo—CoCrMo + P(L,L-L/CL)	4.74	7.85	0.03	0.03
CoCrMo—CoCrMo + P(D,L-L/CL)	5.92	6.94	2.28	1.50

**Table 3:** Results of potentiodynamic tests of resistance to pitting corrosion of CoCrMo alloy modified with biodegradable heparin-releasing coatings exposed to artificial urine for up to 90 days.

Samples	Exposition Time, Days	E_corr_, mV	SD	E_tr_, mV	SD	R_p_, MΩ⋅cm^2^	SD
CoCrMo	0	−72	15	790	7	2.0	0.3
	30	−151	56	775	5	1.3	0.8
	60	−175	100	781	4	1.0	0.5
	90	−101	12	770	1	2.1	0.3
CoCrMo+P(L,L-L/CL)+PVA+HEP	0	+1	10	964	19	6.3	3.4
	30	−145	49	858	37	2.0	0.2
	60	−116	80	828	11	2.2	0.6
	90	−68	50	828	17	2.0	0.4
CoCrMo+P(D,L-L/CL)+PVA+HEP	0	−59	57	955	7	5.8	1.3
	30	−84	9	778	1	1.6	0.1
	60	−147	87	780	2	1.4	0.6
	90	−50	37	783	2	0.8	0.1

E_corr_, corrosion potential; E_tr_, transpasivation potential; R_p_, polarization resistance; SD, standard deviation.

## Data Availability

The data presented in this study are available on request from the corresponding author.
